# Effect of Compression Tape Application on Peri-orbital Ecchymosis After Rhinoplasty: A Prospective, Randomized, Split-face Study

**DOI:** 10.1177/19160216261466650

**Published:** 2026-07-09

**Authors:** Mohammed A. Jomah, Waleed Alshehri, Noémie Villemure-Poliquin, Aseel Doubi, Richard Rival, Oakley Smith

**Affiliations:** 1 Department of Otolaryngology – Head & Neck Surgery, Michael Garron Hospital, University of Toronto, ON, Canada; 2 Department of Otolaryngology – Head & Neck Surgery, College of Medicine, King Saud University, Riyadh, Kingdom of Saudi Arabia; 3 Department of Otolaryngology, Riyadh First Health Cluster, Kingdom of Saudi Arabia; 4 Otorhinolaryngologie et Chirurgie Cervico-Faciale, Faculté de Médecine, Université Laval, Montreal, QC, Canada; 5 Department of Otolaryngology – Head & Neck Surgery, Southlake Regional Hospital, University of Toronto, ON, Canada; 6 Department of Otolaryngology, King Saud Medical City, Riyadh, Kingdom of Saudi Arabia

**Keywords:** rhinoplasty, bruises, ecchymosis, tape

## Abstract

**Importance:**

Postoperative ecchymosis and swelling after open rhinoplasty are commonly encountered and may affect patient satisfaction, aesthetic perception, and recovery. Compression taping has been proposed as a simple technique to reduce these effects, but objective evidence supporting its efficacy remains limited.

**Objective:**

To objectively assess the impact of postoperative compression taping on the extent of ecchymosis in patients undergoing primary open rhinoplasty.

**Design:**

Prospective, split-face, randomized controlled trial.

**Setting:**

Multicenter study in 2 private practice surgical centers over a 4-month period.

**Participants:**

Sixty-four patients undergoing primary open rhinoplasty were enrolled. Exclusion criteria included revision surgeries, obvious facial asymmetry, coagulopathies, and use of anticoagulant medications. One facial side per patient was randomized as the treatment side.

**Intervention or Exposures:**

Compression tape was applied immediately postoperatively to the infraorbital region on the randomized treatment side. The contralateral side received no taping and served as the control.

**Main Outcome Measures:**

The primary outcome was the surface area of ecchymosis measured on postoperative day 5 using standardized digital photography, with quantification performed using Adobe Photoshop software by a blinded assessor.

**Results:**

The study included 64 patients with a mean age of 33.0 years (SD ± 11.3); 48 (75%) were female. The mean ecchymosis surface area on the taped side measured 228.4 mm² (SD ± 120.9) “95% CI 198.8–257.9” compared with 358.9 mm^2^ (SD ± 147.3)) “95% CI 322.9-395.1,” on the control side (*P* < .05), representing a 36.2% reduction in bruising.

**Conclusions:**

Compression taping applied under the eye significantly reduces postoperative ecchymosis following open rhinoplasty. The intervention is simple, safe, and low-cost, supporting its routine use to enhance postoperative recovery.

**Relevance:**

These findings provide objective evidence supporting compression taping as an adjunct postoperative tool to minimize ecchymosis. Future studies may explore its impact on facial swelling, patient-reported outcomes, and broader applications in facial plastic surgery.

## Graphical abstract

**Graphical Abstract:**
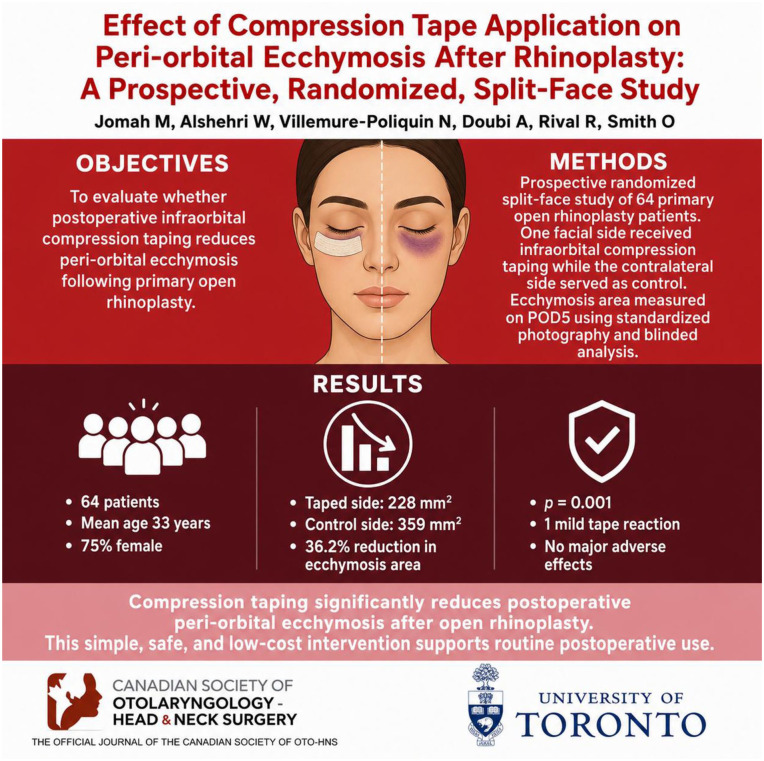
This is a visual representation of the abstract.

## Key Message

1. Compression taping after open rhinoplasty can reduce around 30% of postoperative ecchymosis
2. This study demonstrates a significant benefit from a simple, low-cost intervention.
3. Incorporating compression taping into routine postoperative care may enhance recovery and aesthetic outcomes.

## Introduction

Rhinoplasty is one of the common cosmetic surgical procedures performed. The preoperative consultation for patients seeking rhinoplasty involves discussion of the specific features to be addressed surgically and the frequent undesirable postoperative events, for example, postoperative ecchymosis and swelling. Although these postoperative events are temporary and part of the normal healing process, they might result in a postoperative surgical deformity that leads to considerable distress to the patient both psychologically and physically.^1-3^ The uncertainty of the surgical results in the early postoperative period caused by the masking effect of edema and ecchymosis can cause patient anxiety, social embarrassment, delayed return to work, discomfort, and dissatisfaction.^4^ This can in turn lead to decrease in quality of life, delay in return to normal life activity, and avoidance of social interaction.^4^

Edema and ecchymosis are caused by inflammation and extravasation of blood and interstitial fluid from damaged vessels, which subsequently track through the subcutaneous tissues toward the superficial thin layers of the skin, particularly in the periorbital region.^5^ Multiple techniques have been described to minimize postoperative ecchymosis and swelling.^4^ These include perioperative measures such as the administration of steroid, tranexamic acid, arnica, cold packs application, and head elevation.^3,4,6-9^ Intraoperative measures include controlled hypotensive anesthesia, local injection of vasoconstrictive agent at site of surgery, meticulous atraumatic surgical technique, and avoidance of nasal packing.^4,9,10^ Additionally, it was found that applying tapes over the nose at the end of the procedure helps to decrease nasal edema and ecchymosis in multiple studies.^1,4,9-12^ Taping works as a physical barrier that decreases the blood and fluid accumulation under the lax superficial skin layers.^13^ It is a safe and inexpensive measure that has shown effectiveness in minimizing bruising and swelling.^1,3^ The most common side effect from applying tapes is skin reaction, which is a self-limited, nonserious, and rarely reported event.^4,10-12^ Extending the tapes application to the periorbital region can work in a similar manner to decrease the facial periorbital ecchymosis and swelling. A previously published study found that applying compression tapes under the eye was effective in reducing periorbital edema.^13^ In another study, tapings applied over the upper and lower eyelids were found to be effective in minimizing upper and lower eyelid edema.^11^

Most of the previously published studies for evaluating ecchymosis post rhinoplasty utilized subjective measurement scales that depend on human rater judgment.^4^ The subjective measurement scales are prone to bias and largely depend on rater knowledge and experience.^4^ In addition, rater consistency and inter-rater agreement might affect the standardization and make it difficult to make comparisons between different studies.

The purpose of this study was to evaluate the effect of infraorbital compression taping on postoperative ecchymosis following rhinoplasty. We hypothesized that compression taping would act as a physical barrier to limit the spread of ecchymosis and thereby reduce its visibility. The specific aim of this study was to quantitatively assess the surface area of ecchymosis using high-resolution digital photographs and to compare the taped side with the contralateral nontaped side of the face.

## Methods

### Study Design and Sample

The study was reviewed and approved by the Research Ethic Board in Michael Garron Hospital, University of Toronto, Toronto, ON. Ethical approval (Reference No. 881-2210-Mis-389) and informed written consents were obtained from all the participants.

We conducted a prospective split-face controlled trial including all adult patients who underwent primary open rhinoplasty between March 2024 and June 2024 at 2 private clinics located in Toronto, ON, Canada (Beach Cosmetic Clinic and Rival Cosmetic Surgery Clinic). Patients having revision surgery, or those with significant facial asymmetry, coagulopathies, or receiving anticoagulant therapy were excluded from the study.

### Predictor Variable

The predictor variable was postoperative application of compression taping. For each patient, one side of the face was randomly selected to receive compression taping of the infraorbital region, while the contralateral side served as the control without taping.

### Outcome Variable

The primary outcome variable was the surface area of peri-orbital ecchymosis, measured on postoperative day 5.

### Covariates

The covariates included age, sex, surgeon, and osteotomy technique (piezoelectric vs conventional).

### Randomization

Randomization was performed using a computer-generated list created in Microsoft Excel (Mac OS). Odd numbers designated the right side of the face for taping, and even numbers designated the left side. Allocation was concealed and the list was kept with a third party, and the assigned number for each patient were revealed to the surgeon after completion of the surgical procedure and skin closure.

### Surgical Procedure and Intervention

All surgical procedures were performed by 2 certified facial plastic surgeons (OS, RR). All rhinoplasties were performed using an open approach with supra-perichondrial dissection at the lower vault and sub-periosteal dissection at the upper vault. All patients underwent open septoplasty after tip-splitting. Upper and mid-vault modifications, tip-plasty, and dorsal reduction were individually tailored. Dorsal reduction was performed using a fine osteotome, followed by minimal rasping when required. Nasal tip refinement included a cephalic trim, preserving at least 8 mm of the lower lateral cartilages. Tip suturing techniques varied according to the anatomy, including domal creation with interdomal sutures and either medial or lateral crural steal, depending on tip position and lower lateral cartilage length. A tongue-in-groove maneuver was employed in nearly all cases, while some patients required a septal extension graft harvested from the nasal septal cartilage. Bilateral paramedian and lateral low-low osteotomies were performed in all patients. A piezo-electric device was used for osteotomy by 1 surgeon (OS), while conventional osteotomies by curved guided osteotome were utilized by the other surgeon (RR). No additional functional nasal procedures were performed. General hypotensive anesthesia with endotracheal intubation, in addition to local anesthesia (Lidocaine 1% + Epinephrine 1:100 000) infiltration were used in all cases. Preoperative oral steroid (Prednisolone 50 mg PO for 2 days) and perioperative tranexamic acid (TXA 1 gm PO Daily for 5 days started 1 day before surgery) were given to all patients.

**Figure 1.**
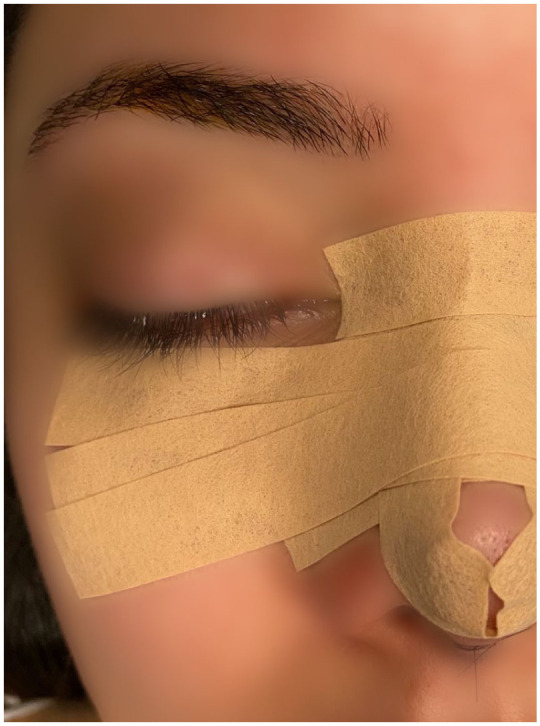
A photo demonstrating the placement of taping under the eye on the intervention side of the face.

After the completion of the surgical procedure, the nasal tapes and splint were applied as the standard of care for all patients. Compression tapes (3M Micropore 0.5 Inch tapes) were tightly applied under the eye over the cheek extending from the lateral edge of the nose till the lateral malar region on the intervention side of the face (Figure 1). The other side of the face was used as a control and no tapes were applied. The tapes were kept on till the first postoperative visit at day 5, where tapes and cast were removed, and a digital photograph of the patient was taken immediately for analysis. All photos were taken in the same conditions using Nikon D3100 camera with a 35 mm lens at fixed distance and height with wall-mounted lights.

### Data Collection Methods

Ecchymosis surface area measurements were performed by an assessor (WA) who was blinded to the intervention side. Adobe Photoshop software for Mac (25.9.1) was used for the measurement. The edges of the area of ecchymosis (yellow, blue, and purple discoloration) were carefully marked on the digital photos, and surface area was calculated by converting surface area in pixel to millimeters (1 pixel = 0.07 mm). The width of the iris (considered as 10 mm in diameter) in each patient was used as a reference for standardization of the measurements.^11^

### Sample Size

The sample size was calculated based on the expected difference in means and standard deviations derived from previously published studies.^11^ The required sample size was 44 participants per group. To account for an anticipated 15% loss to follow-up, this number was increased to 50 per group. Given that each participant served as their own control, and assuming a two-sided significance level of 0.05 with 80% power, a total of 50 participants was required.

### Data Analyses

SAS University Edition software was used for statistical analysis. Categorical variables were presented as proportions with percentages, and continuous variables were presented as means with standard deviations (SD). The differences in bruising surface area between the intervention side (with taping) versus the control side (without taping) was calculated using a Wilcoxon signed-rank test and presented as a mean difference, as well as a mean percentage of reduction. The significance level for all statistical tests was set at a = 0.05, indicating that results with a *P*-value less than .05 were considered statistically significant.

## Results

**Figure 2.**
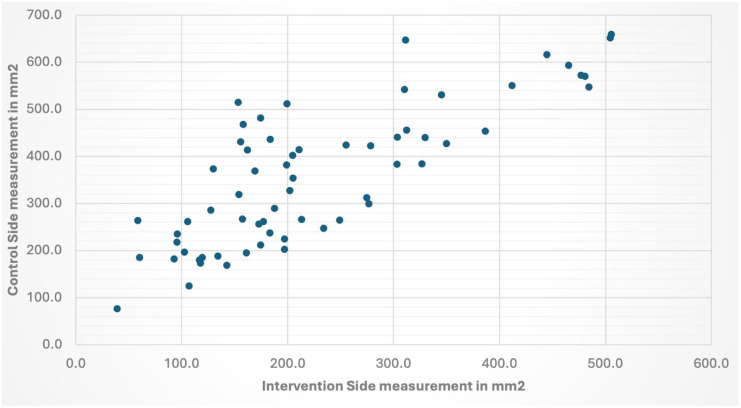
Scatter plot for surface area measurements in intervention and control sides of each patient.

A total of 64 patients were included in the study, with 16 males and 48 females. The mean age was 33.04 years (SD 10.7) “95% CI 29.8-36.2” (Table 1). All patients completed the follow-up and were assessed on the 5th day postoperatively. Figure 2 shows the measurement of the 2 sides for each participant in the study.

**Table 1.** Participant Demographics With the Ecchymosis Surface Area Measurement.

Total Number of Patients	Age mean (SD) “95%CI”	Gender Male: Female	Control Side Surface Area mm^2^ Mean (SD) “95%CI”	Intervention Side Surface Area mm^2^ Mean (SD) “95%CI”	Difference Between Sides Mean (SD) “95%CI”	*P*-value	Average % of Reduction in Ecchymosis Surface Area
64	33.04 (10.7) “29.8-36.2”	16:48	358.9 (147.25) “322.9-395.1”	228.4 (120.9) “198.8-257.9”	130.6 (87.83) “109.1-152.1”	.001	36.20%

The mean surface area for the intervention side (with taping) was 228.4 mm^2^ (SD 120.9) “95% CI 198.8 to 257.9,” while for the nontape side it was 358.9 mm^2^ (SD 147.3) “95% CI 322.9-395.1.” The difference between the 2 sides was statistically significant with a *P*-value <.05. The mean surface area difference between the 2 sides was 130.61 mm^2^ “95% CI 109.1 to 152.1,” showing a 36.2% reduction in the ecchymosis surface area on the intervention side (Table 1).

Subgroup analysis between the 2 different osteotomy technique groups showed no statistically significant difference in the mean surface area difference. The mean surface area differences between the 2 sides for the surgeons, OS and RR, were 138.1 mm^2^ (SD 95.5) “95% CI 165.1 to 111.1” and 108.1 mm^2^ (SD 55.7) “95% CI 135.4 to 80.8,” respectively, indicating no significant effect of the osteotomy technique (ie, piezoelectric vs conventional osteotomy) on the reduction of ecchymosis surface area.

There was one case that developed an allergic reaction to the Steri-strip tape in the form of localized, limited swelling, which resolved spontaneously after the removal of the tapes. No other side effects were encountered in the other patients.

## Discussion

Postoperative ecchymosis and edema are common events following rhinoplasty. The appearance of these undesirable features leads to patient concerns and hinders early social interactions. Ecchymosis occurs when small blood vessels break due to the force exerted during surgery, leading to the extravasation of blood into the surrounding connective tissue. This typically activates an inflammatory response, where neutrophils and macrophages infiltrate the area, aiding in the healing process.^14^ The extent of bruising depends on factors such as the degree of tissue damage, blood pressure, skin color and thickness, and proximity of the bleeding source to the skin’s surface.^14,15^ The area between the dermis and hypodermis, where loose connective tissue is located, allows for more extensive extravasation.^14^ The periorbital region is characterized by scanty connective tissue content and rich adipose and vascularity contents, which contribute to the increase of ecchymosis appearance.^15^

The appearance of ecchymosis usually diminishes within days with a natural course following a distinct pattern. Initially, within the first 24 hours, bruises typically present as red or blue due to the presence of hemoglobin in extravasated red blood cells.^14,15^ By the third to fifth day, the color begins to change from blue to brown, reflecting the breakdown of hemoglobin into hemosiderin, a brownish pigment.^14,15^ Histologically, hemosiderin-laden macrophages become prominent at this stage. Additionally, hematoidin, a yellow-brown pigment, begins to form extracellularly as the bruise progresses toward healing.^14,15^ This corresponds with the later stages where the color shifts to yellow green, indicating the resolution phase.^14,15^ However, this sequence of color change has been found to be unreliable for accurate determination of ecchymosis age and multiple factors can contribute to variations in ecchymosis appearance between individuals.^15^

Several modalities have been identified to reduce periorbital ecchymosis after rhinoplasty. The use of perioperative corticosteroids, especially dexamethasone, is the most extensively studied and has shown significant efficacy in decreasing both edema and ecchymosis postoperatively.^6^ In addition, intraoperative interventions such as maintaining hypotension and using cold compresses have demonstrated positive effects in reducing bruising. Another key factor is postoperative care, where head elevation and ice application have proven to be highly effective in reducing periorbital swelling and discoloration. Surgical techniques like limiting periosteal elevation prior to osteotomy and meticulous tissue handling also help minimize these postoperative morbidities. While the literature presents mixed results on herbal supplements, some studies have shown that Arnica and bromelain may offer additional benefits with minimal risk.^3,4,10^

The application of tapes after rhinoplasty plays a supportive role in minimizing postoperative ecchymosis. Taping helps to provide mild compression, acting as a physical barrier, which stabilizes the tissues, reduces the spread of fluid into the periorbital area, reduces the pooling of blood under the skin, and promotes the drainage of blood and interstitial fluid.^9^ This results in a reduction of ecchymosis and swelling around the eyes, contributing to faster recovery and better aesthetic outcomes.^9^ In addition, tape application can reduce patient anxiety and stress by hiding the appearance of bruises.^1,3^

Few studies have investigated the effect of taping in reducing ecchymosis after rhinoplasty. Farahvash et al. reported a significant reduction in the ecchymosis surface area and patient satisfaction at day 7 postoperatively with the use of compression tape under the eye.^11^ In another study, Tatar et al. reported a reduction in bruising surface area at day 3 postoperatively when compression taping was applied over the upper and lower eyelids.^12^ Our results show a similar positive effect of compression tape application on the fifth postoperative day, consistent with these previous findings. Additionally, our multicenter cohort, involving procedures performed by 2 board-certified surgeons, demonstrates that compression taping exerts a beneficial effect regardless of the osteotomy technique used, whether piezoelectric or conventional.

In our study, we quantitatively assessed the amount of reduction in ecchymosis surface area after the application of tapes. We kept the tapes on for 5 days, which are a typical duration for suture and cast removal. In addition, the 3rd to 5th postoperative days represent the peak of ecchymosis visibility.^14,15^ At this stage, most patients in our study exhibited faint yellowish ecchymosis on both sides of the face. The primary difference was in the surface area affected, while the coloration remained consistent. None of the patients reported a difference in pain or discomfort between the 2 sides of the face. Our results showed that the use of a piezo-electric device for osteotomies did not result in a significantly different amount of ecchymosis compared to conventional osteotomy. However, this difference could be attributed to the surgical skills of the 2 senior surgeons.

Our study is limited by a small sample size and a relatively short follow-up period. Additionally, patient-reported outcomes, such as satisfaction and aesthetic perception, were not assessed through a formal survey, which may have provided further insight into the clinical impact of the techniques used. Future studies of compression tapes would benefit from incorporating validated patient-reported outcome measures, including visual assessment methods in which mirror images of the taped and untaped sides of the face are shown to patients, who are then asked to assess and identify the side with the more favorable outcome.

In conclusion, the application of tapes under the eyes is a safe and cost-effective method for reducing periorbital ecchymosis. Our results confirm the positive effect of taping reported in previously published studies.
